# *Journal of Educational Evaluation for Health Professions* received the top-ranking Journal Impact Factor―9.3—in the category of Education, Scientific Disciplines in the 2023 Journal Citation Ranking by Clarivate

**DOI:** 10.3352/jeehp.2024.21.16

**Published:** 2024-06-24

**Authors:** Sun Huh

**Affiliations:** Institute of Medical Education, College of Medicine, Hallym University, Chuncheon, Korea; Hallym University, Korea

I was thrilled to discover that the Journal Impact Factor (JIF) of our journal—*Journal of Educational Evaluation for Health Professions* (JEEHP) reached 9.3 in the 2023 Journal Citation Ranking by Clarivate, marking it as the highest in the category of Education, Scientific Disciplines on June 20, 2024 (Fig. 1). To commemorate this remarkable achievement, I would like to reflect on the 20 years of my editorship in this editorial.

## Launch of the journal in 2004

**Fig. 2.**
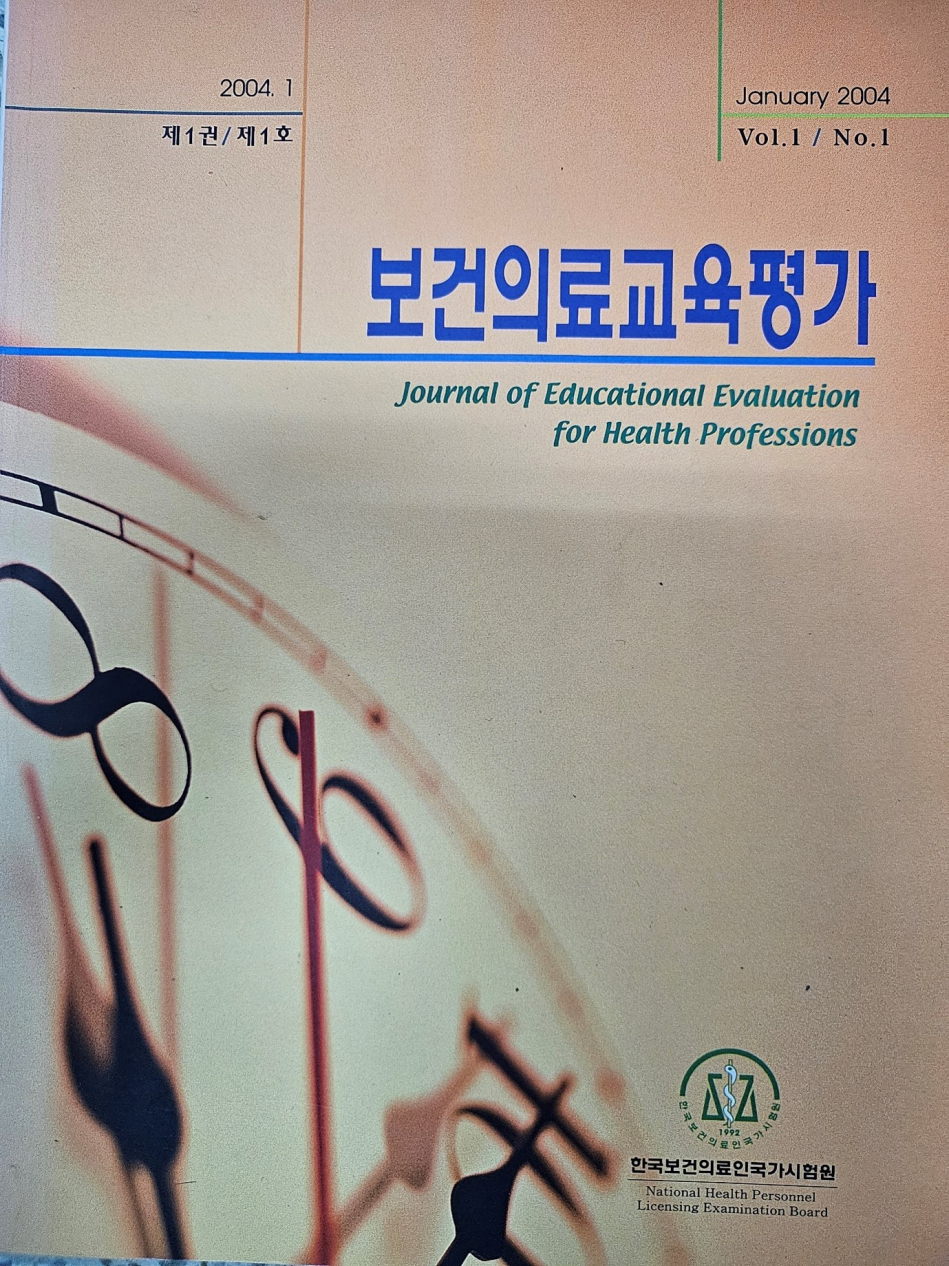
Photo of the cover of the inauguration issue of the *Journal of Educational Evaluation for Health Professions*.

**Fig. 3.**
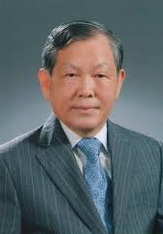
Photo of Dr. Sang-Ho Baik, the 2nd president of the Korea Health Personnel Licensing Examination Institute (May 8, 2001–May 7, 2004).

**Fig. 4.**
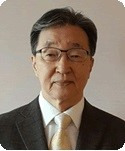
Photo of Dr. Chan-Il Park, the inaugural editor of the *Journal of Educational Evaluation for Health Professions*.

This year marks the 20th anniversary of the JEEHP (Fig. 2), which was launched in 2004 at the behest of Dr. Sang-Ho Baik, the then-president of the Korea Health Personnel Licensing Examination Institute (formerly known as the National Health Personnel Licensing Examination Board) (Fig. 3). Dr. Chan-Il Park of the Department of Pathology at Yonsei University College of Medicine (Fig. 4) edited and published the inaugural issue, which included 9 significant articles on educational evaluation. These articles have proven to be valuable resources for instructors in the field of healthcare education in Korea. The inaugural issue was expertly edited, and all nine papers continue to be cited to this day. Among these, I contributed a paper that analyzed the results of the Korean Medical Licensing Examination using item response theory [1].

In 2005, following Dr. Park’s recommendation, I took on the editorship of this journal and have been in the role for 20 years. I distinctly recall the words of someone who declined the same role, stating, “This journal is trash.” Similarly, Dr. Dae-Myung Jue, a renowned editor in Korea, once heard a library director describe all Korean domestic journals as “garbage bins.” Despite this, Dr. Jue successfully elevated his journal, *Experimental and Molecular Medicine*, to one of the top ranks in the molecular medicine and biochemistry category [2]. However, I accepted the editorship without hesitation, believing it inappropriate for a junior to reject an offer from a respected senior. At the time, Professor Park was highly esteemed as a leading editor in Korea, and I could not have envisioned serving as editor for 2 decades.

## Plan for development into an international journal

After publishing the second issue, which contained 9 Korean articles in December 2005, I began to question the sustainability of continuing in this manner over the long term. Observing that all leading companies in Korea, like Samsung, Hyundai, and LG, survive by engaging in import and export activities within the global market, I concluded that JEEHP, as a specialized journal, would also face difficulties if it continued to cater exclusively to Korean researchers as both authors and readers. This concern was compounded by the scarcity of experts in this field within Korea and the fact that the journal was newly established and not yet indexed in international databases.

The first step toward becoming an international journal was transitioning to an English-language format in 2006, with the goal of being indexed in PubMed Central (PMC). At that time, journals that submitted PMC XML files were nearly all accepted for inclusion in PMC [3]. After successfully producing PMC XML files in July 2006, I implemented them on the journal’s website. I then applied for inclusion in PMC and was accepted in February 2009. This PMC indexing marked a groundbreaking event in the history of Korean medical journals. By introducing PMC XML production to several journals, a wave of domestic journals transitioned to English and became indexed in PMC. My efforts to develop JEEHP into an international journal initiated a movement that significantly impacted the advancement of Korean medical journals. In addition to PMC indexing, since 2007, I have been assigning DOIs and depositing Crossref XML files, fully utilizing various Crossref services [4].

## Increased submissions after being indexed in international databases

Even after JEEHP was indexed in PMC, there was no significant increase in domestic submissions., However, submissions from abroad did increase. In 2015, we published a total of 58 articles, comprising 10 domestic papers and 48 papers from 18 different countries. Subsequently, due to budget constraints, we adjusted the publication volume from 58 articles to approximately 40 articles per year. Therefore, the acceptance rate of unsolicited manuscripts gradually decreased from 28.6% in 2016 to 11.9% in 2023 due to the increased number of submissions and limited space.

Among international journals in the field of healthcare education, JEEHP is distinguished by its unique focus on educational evaluation. This unique scope led to the journal being indexed in MEDLINE [5] and the Emerging Sources Citation Index (ESCI) in 2016, EMBASE in 2018, and Scopus in 2019 [6]. In 2023, the 2022 JIF reached 4.4, ranking 7th among 85 SCIE and ESCI journals in the category of Education, Scientific Disciplines [7]. The 2023 JIF, 9.3, ranks 1st among 85 SCIE and ESCI journals.

## What does it mean to be the top-ranking journal in the category of Education, Scientific Disciplines

This achievement illustrates that a local institutional journal can reach the highest standards without the backing of international commercial publishers like Elsevier, Springer-Nature, and Wiley-Blackwell, solely through dedicated efforts. The Korea Health Personnel Licensing Examination Institute stands as a global leader in managing healthcare licensing examinations. In line with its prominence, the institute aims for its journal to reflect the highest quality, a goal shared by its members and all professionals involved in its operations. However, the journal lacks dedicated full-time staff, presenting significant challenges in competing with international commercial publishers, especially in terms of budget, editing, and publication.

However, with the support of editorial board members and peer reviewers from around the world, along with the superior capabilities of Korean publishing companies such as InfoLumi for manuscript editing (https://infolumi.co.kr/), M2PI for digital publication (https://www.m2-pi.com/), Research Factory for graphical abstracts (https://www.rfactory.kr/), and Compecs for English proofreading (https://www.compecs.com/), the journal has achieved international standards. Despite budget constraints that limit spending, the staff from these companies have delivered top-notch services, allowing us to maintain an international publication standard without compromising quality. In the realm of online publishing, the creation of high-quality JATS XML files and their conversion to other XML formats is essential [8], and this task has been adeptly handled by M2PI. For the digital standards of scholarly journal publishing, Crossref services, including digital object identifier, check for updates, cited-by functions, funder registry, similarity check, metadata retrieval, and participation reports, are other essential parts [9], which have also been excellently provided by M2PI. While the editorial board and reviewers are crucial in selecting articles, without technical support, proper journal formatting would be unachievable. I am proud to collaborate with these leading publishing companies.

## How to maintain the journal’s quality

Some may question whether the JIF of 9.3 could potentially be due to chance. It is true that in the coming year, it is not guaranteed that such a high score will be achieved, as high citation counts often rely on a few standout articles (Table 1).

Since achieving a higher JIF is not an explicit goal of the journal, it is crucial to focus more intensively on topics relevant to health professionals and their students. As we look to the future, marked by the emergence of artificial intelligence, 6G-based internet, quantum computing, and humanoid robots, predicting the evolution of healthcare education and educational evaluation becomes increasingly complex. It is equally challenging to determine the appropriate topics for JEEHP to maintain its esteemed reputation. To remain competitive with international commercial publishing companies, we must train editing experts who are equipped to adapt to these changes and prepare for the future of healthcare education and the journal.

Another task ahead involves identifying and supporting researchers who can submit research reports funded by the Korea Health Personnel Licensing Examination Institute and formatted according to JEEHP’s guidelines. By sharing these valuable research findings with healthcare educators worldwide through JEEHP, our journal will undoubtedly establish itself and thrive as the most practical source of information in the field of healthcare licensing examinations worldwide.

## Appreciation to authors, reviewers, volunteers, and company staff

The accomplishments of JEEHP to date are the result of the dedication and passion of researchers who have generously shared their valuable findings, both past and present employees of the Korea Health Personnel Licensing Examination Institute, and past and present editorial board members who have meticulously edited and published manuscripts to meet international standards. Additionally, the staff of the publishing companies who collaborated with us have played a crucial role. I am deeply thankful to everyone who has patiently supported and waited for the publication of the journal, helping it achieve its current status.

## Figures and Tables

**Fig. 1.**
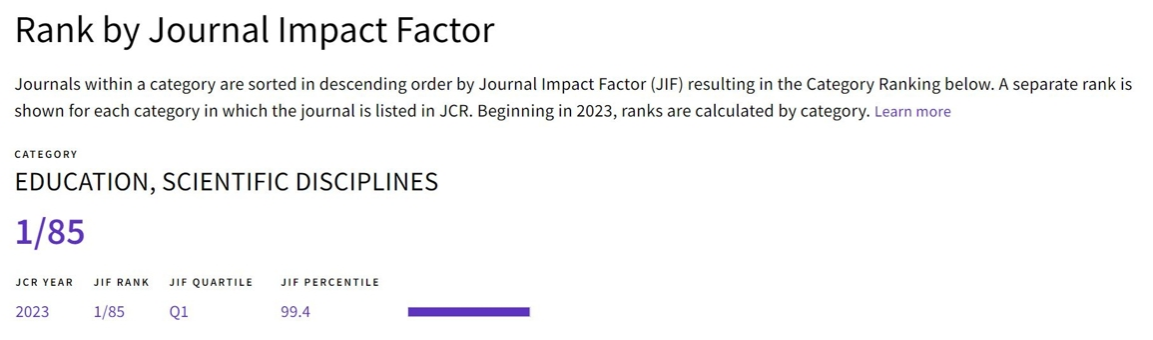
The 2023 Journal Citation Ranking results in the category of Education, Scientific Disciplines.

**Table 1.** Top 5 highly cited articles published in 2021–2022 and cited in 2023 in the Web of Science Core Collection

Rank	Article title	Year	No. of citations
1	Sample size determination and power analysis using the G*Power software	2021	283
2	Educational applications of metaverse: possibilities and limitations	2022	131
3	E-learning in health professions education during the COVID-19 pandemic: a systematic review	2021	38
4	Training in lung cancer surgery through the metaverse, including extended reality, in the smart operating room of Seoul National University Bundang Hospital, Korea	2021	33
5	Application of computer-based testing in the Korean Medical Licensing Examination, the emergence of the metaverse in medical education, journal metrics and statistics, and appreciation to reviewers and volunteers	2022	15

COVID-19, coronavirus disease 2019.
